# Evaluating Participation in Gender-Affirming Care: Cross-Sectional Analysis of Dermatology Program Websites in the United States

**DOI:** 10.2196/54480

**Published:** 2024-02-12

**Authors:** Marco Costanza, Jeffrey Sobieraj, Frank Wang

**Affiliations:** 1 University of Michigan Medical School Ann Arbor, MI United States; 2 Department of Dermatology Michigan Medicine University of Michigan Ann Arbor, MI United States

**Keywords:** dermatology, gender-affirming care, transgender, dermatology residency, medical education, website, digital platform, media, gender, websites, school, resident, residencies, residency, schools, universities, universities, cross-sectional

## Introduction

Transgender and gender-diverse (TGD) patients have unique dermatologic needs, including management of complications from gender-affirming hormone therapy or surgery [1]. Dermatologists play a pivotal role addressing these needs and providing services for gender-affirming care (GAC), such as laser hair removal, management of androgenetic alopecia, injectable neurotoxins, or soft tissue augmentation. To ensure culturally competent care, dermatology residency programs should provide/promote didactic and experiential training tailored to the health needs of TGD patients [2]. Additionally, prospective residents may benefit from being able to ascertain whether certain programs are involved in GAC, including education and research. We aimed to assess the current landscape of GAC participation among dermatology programs and propose strategies to enhance the visibility of such participation.

## Methods

Using Doximity 2022-2023 Residency Navigator, dermatology residency programs were identified (N=141). From April to July 2023, the websites of each department, residency program, and associated institution were examined to identify participation in GAC. Next, web-based searches were conducted using department and residency program names plus the following terms: “LGBTQ health,” “gender affirming care,” “transgender healthcare,” or “transgender.” Search results were used to identify institutional multidisciplinary GAC programs, volunteer-based services/clinics participating in GAC, and participation in GAC not otherwise mentioned on program websites. Programs were independently reviewed and categorized by authors MC and JS. Interrater reliability was calculated using Cohen κ. Scores ≥0.8 were considered acceptable [3]. For discrepancies in categorization, searches were reconducted with the results discussed to reach a consensus.

## Results

**Figure 1:**
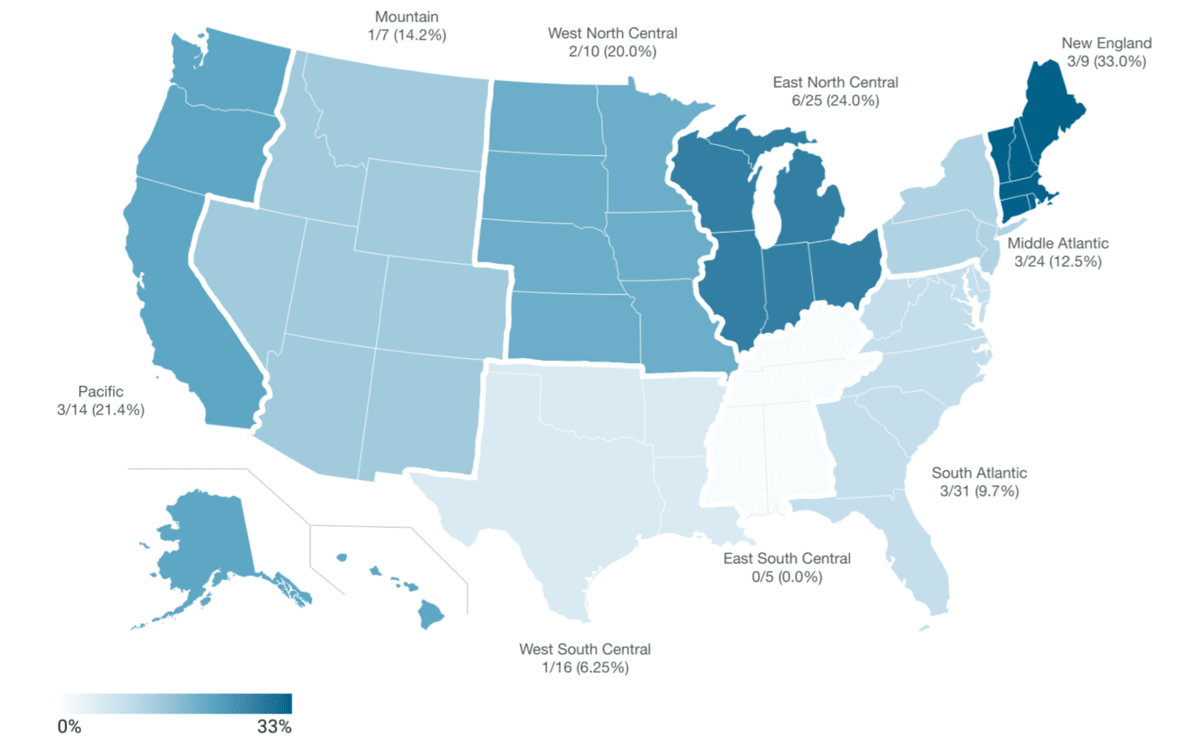
Geographic distribution of dermatology programs participating in gender-affirming care. The choropleth map shows the proportion of programs involved in gender-affirming care (GAC) per geographic region, as defined by the Association of American Medical Colleges. The number of programs with GAC out of total programs in the region are displayed, along with associated percentages and color according to the scale.

Among the 141 examined websites, we found that 22 (15.6%) dermatology programs mentioned providing GAC; the type of participation was variable (Table 1). The remaining programs (n=119, 84.4%) did not mention participating in dermatologic GAC. Of this group, 62 were part of institutions with multidisciplinary GAC programs, while 57 were not. Among the 22 programs participating in GAC, geographic distribution was variable, with the greatest number in the New England region (Figure 1).

**Table 1:** US dermatology residency programs mentioning involvement in gender-affirming care (GAC).

			Programs (N=141), n (%)
**Mentioning participation in GAC**			22 (15.6)
	Participation in an institutional multidisciplinary GAC clinic^a^	22 (100.0)	
	Listing a directory of SGM^b^ health providers	19 (86.4)	
	Listing specific gender-affirming dermatologic procedures (eg, electrolysis or neurotoxins)	12 (54.5)	
	Listing GAC under a “services offered” tab	6 (27.3)	
	GAC program led by dermatology department	3 (13.6)	
**Not mentioning participation in** **GAC**			119 (84.4)
	Multidisciplinary GAC clinic at institution but no mention of dermatology involvement	62 (52.1)	
	No mention of gender-affirming care on institutional website	57 (47.9)	

^a^Participation in a multidisciplinary clinic was defined as at least one faculty member representing the department in the clinic.

^b^SGM: sexual and gender minority.

## Discussion

We found that a minority of dermatology programs mentioned participating in GAC, indicating that there remains a considerable gap between the desired and current state of resident education in gender minority health [4,5]. Indeed, dermatology residents receive, on average, 75 minutes of sexual and gender minority (SGM) health education yearly [5] and report low competency and confidence in caring for TGD patients [5,6]. Furthermore, dermatology program directors report barriers to implementing SGM health training, such as lack of funding, curricular time, and experienced faculty [4].

We observed that over 60 dermatology programs did not mention participating in GAC but are affiliated with institutions with multidisciplinary GAC clinics. These programs may consider collaborating with providers in those clinics to improve resident education and care of TGD patients. Highlighting such collaborations may aid recruitment of SGM-identifying residency/faculty candidates, especially those interested in teaching or studying SGM dermatology.

Furthermore, it is possible that some programs actually participate in GAC but do not “advertise” it on websites. Importantly, scrutiny or legal repercussions may affect the visibility or availability of GAC services of some programs, particularly those affiliated with pediatric hospitals. Thus, when permissible, programs can implement simple measures to highlight their efforts. Program websites could identify departmental or institutional providers passionate about providing GAC. Programs may provide information on whether they perform minimally invasive procedures for GAC, like laser hair removal, injectable neurotoxins, or soft tissue augmentation. Likewise, displaying images of providers wearing pronoun badges or “pride pins” may foster an inclusive environment for patients and providers [7]. These measures do not require curricular time or funding and are associated with improved health outcomes [1,7].

Overall, our results expand upon those of a recent study, specifically by indicating how dermatology programs participate in GAC beyond involvement in multidisciplinary clinics [8]. Our study’s limitations include using publicly available websites, which may not fully reflect TGD health content within curricula, collaborations with GAC experts, or dermatology research related to TGD patients. Future research can address these limitations by surveying program directors or multidisciplinary GAC clinics to ascertain the specifics of departmental involvement.

Our study provides insights into the various types of participation in GAC among dermatology residency programs, as well as existing challenges program directors face and potential clinical and nonclinical opportunities for improvement. Program websites may serve as a valuable and accessible resource to help TGD patients obtain GAC and to attract diverse residency and faculty candidates to a program. To cultivate a safe environment for patients and providers alike, program directors could consider, when possible/permissible, relatively easy yet impactful ways to use their program/departmental websites to enhance and advertise their participation in GAC.

## Abbreviations

GAC: gender-affirming care
SGM: sexual and gender minority
TGD: transgender and gender-diverse
